# The study of neural antibodies in neurology: A practical summary

**DOI:** 10.3389/fimmu.2022.1043723

**Published:** 2022-12-07

**Authors:** Mireya Fernández-Fournier, Laura Lacruz, Pilar Nozal, Juan Luis Chico, Antonio Tallón Barranco, Laura Otero-Ortega, Iñigo Corral, Angela Carrasco

**Affiliations:** ^1^ Neuroimmunology and MS Unit, Department of Neurology, La Paz University Hospital, Neurology and Cerebrovascular Disease Group, Neuroscience Area of Hospital La Paz Institute for Health Research – IdiPAZ, Universidad Autónoma de Madrid,Madrid, Spain; ^2^ Department of Immunology, La Paz University Hospital, Complement Research Group, of Hospital La Paz Institute for Health Research – IdiPAZ, Center for Biomedical Network Research on Rare Diseases (Ciberer), Madrid, Spain; ^3^ Department of Neurology, Ramon y Cajal University Hospital, Instituto Ramón y Cajal de Investigación Sanitaria IRYCIS, Madrid, Spain; ^4^ Neurology and Cerebrovascular Disease Group, Neuroscience Area of Hospital La Paz Institute for Health Research – IdiPAZ (La Paz University Hospital- Universidad Autónoma de Madrid), Madrid, Spain; ^5^ Department of Immunology, Ramon y Cajal University Hospital, Instituto Ramón y Cajal de Investigación Sanitaria IRYCIS, Madrid, Spain

**Keywords:** neuroimmunology, antibodies, cell surface antigens, intracellular antigens, laboratory techniques

## Abstract

The field of Autoimmune Neurology is expanding rapidly, with new neural antibodies being identified each year. However, these disorders remain rare. Deciding when to test for these antibodies, when and what samples are to be obtained, how to handle and study them correctly, and how to interpret test results, is complex. In this article we review current diagnostic techniques and provide a comprehensive explanation on the study of these patients, in an effort to help with correct diagnosis minimizing false positive and false negative results. We also propose routine storage of samples and referral of certain cases to specialized research laboratories.

## Introduction

Neuroimmunology is an expanding field of research. The identification in recent decades of new autoantibodies against target antigens both in neurons and glial cells has permitted to characterize cases of previously unexplained neurological disease. However, the expansion of this field and the fact that seronegative forms that respond to immunomodulatory therapy (1) are diagnosed suggests that there are still clinical syndromes and antibodies to be characterized.

The finding of neural antibodies should always be considered in the context of the patient’s symptoms (2, 3). The presence of an antibody does not always define immune-mediated neurological disease and may occur due to other conditions of the patient (as occurs, for example, with anti-GAD 65 antibodies) (4). Likewise, the presence of some of these autoantibodies has been described in cancer patients without neurological disease (5). Also, presence in serum but not in cerebrospinal fluid (CSF) may mean that the detected antibody is not mediating the patient’s neurological symptoms.

Antineuronal autoantibodies can be directed against intracellular antigens or against cell surface proteins, many are located behind the blood-brain barrier where they present intrathecal synthesis of antibodies by plasma cells in the brain or in the meninges (6). Each antibody presents a characteristic immunohistochemical staining pattern, however the study and interpretation of the results of the different techniques can be complex, it requires obtaining and handling of appropriate samples correctly, as well as training to identify the different patterns.

The very low incidence of these diseases further contributes to make the diagnostic process difficult and makes the study in non-specialized laboratories complex. To offer the best care and diagnosis to patients, it may be useful to establish working networks that can help detect new cases through a correct identification of antibodies already characterized or not, through the study of patient samples on neural tissue by immunofluorescence or immunohistochemistry (7) and facilitate the communication between local laboratories with referral laboratories. This may be helpful in order to improve antibody testing not available by commercial assays. It is convenient to address and standardize working procedures throughout local laboratories, which may then form collaborative networks with specialized referral centres.

## When to study neural antibodies

Patients should be studied for neural antibodies if they present a neurological syndrome typically associated with these antibodies (8–11):

1. Limbic encephalitis and encephalitis fulfilling criteria for possible autoimmune encephalitis.
2. Encephalomyelitis.
3. Brainstem encephalitis.
4. A rapidly progressive cerebellar syndrome.
5. Opsoclonus-myoclonus.
6. Sensory neuronopathy.
7. Axonal polyradiculoneuropathy of subacute onset.
8. Demyelinating polyradiculoneuropathies with a prolonged subacute phase or poor treatment response
9. Stiff-person or Morvan syndrome.
10. Lambert-Eaton myasthenic syndrome.
11. Gastrointestinal pseudo-obstruction (enteric neuropathy).
12. Faciobrachial dystonic seizures, temporal lobe epilepsy of unknown origin or refractory epileptic seizures.
13. Other neurological syndromes of subacute onset (<3 months) and with inflammatory findings in CSF or MRI that are suggestive of a possible autoimmune origin (having ruled out other causes including infectious, metabolic, tumour etc.)

Of note, immune-mediated encephalitis accounts for a substantial proportion of encephalitis cases and is among the most important differential diagnoses for rapidly progressive dementia (RPD) (12). However, antineuronal antibodies are listed as “Secondary Tier” (depending on initial screen and clinical scenario) and not as initial screening for cases of RPD (13).

Subacute sensory neuronopathy was the first and most frequently observed peripheral PNS, but, as described in the literature, the spectrum of has increased to encompass motor neuropathies, small fiber neuropathies, and autonomic as well as nerve hyperexcitability syndromes. Of note, also focal neuropathies, as cranial nerves, plexopathies, and mononeuropathies, are considered in some cases to be of paraneoplastic origin (9). Even if paraneoplastic polyradiculoneuropathies are more commonly axonal, non-paraneoplastic demyelinating polyradiculoneuropathies may be associated with the presence of neural antibodies against nodal or paranodal proteins, specially those with a prolonged subacute phase or poor treatment response, and this should be investigated in these cases (14, 15).

Regarding refractory epileptic seizures, both focal and generalized tonic-clonic, especially when repetitive and of short duration, or associated with dysautonomic symptoms, particularly piloerection (16) or abnormal perceptual phenomena (déjà-vu, autoscopy…) should raise suspicion for antineuronal antibody associated disease. Especially those occurring in patients without previous diagnosis of epilepsy, but not limited to these patients.

The treating physician should indicate which autoantibodies must be analysed after considering the patient’s clinical presentation (including imaging and other laboratory tests). Given the diagnostic complexity of these disorders due to heterogeneous presentations, where the same antibody can result in a spectrum of different manifestations and a clinical syndrome can be associated with different autoantibodies (17), and given the training required for a correct and complete neurological evaluation, it is recommended that all cases be evaluated by a certified neurologist with experience in Neuroimmunology.

As pointed out previously, indiscriminate and unfocused testing increases the chances of false-positive and false-negative results (9).

## Laboratory work-up

### Procedures

Antineuronal antibodies are classified into antibodies against intracellular antigens (frequently of paraneoplastic origin) and those against neuronal surface antigens. Validated screening techniques for both type of antibodies are indirect immunofluorescence (IIF) on mouse cerebellum, hippocampus and nerve tissue, and immunohistochemistry (IHC) on rat cerebellum and hippocampus tissue (18). Even if both techniques use cerebellum and hippocampus tissue, they differ in tissue preparation. For IHC, rats must be pre-treated with paraformahalide and cryopreservation performed. Thus, specialized personnel is needed. In the case of IIF, tissues are fixed on slides without prior treatment and the standard technique is performed with fluorochrome for antibody identification. However, immunofluorescence decays rapidly over time.

**Figure 1:**
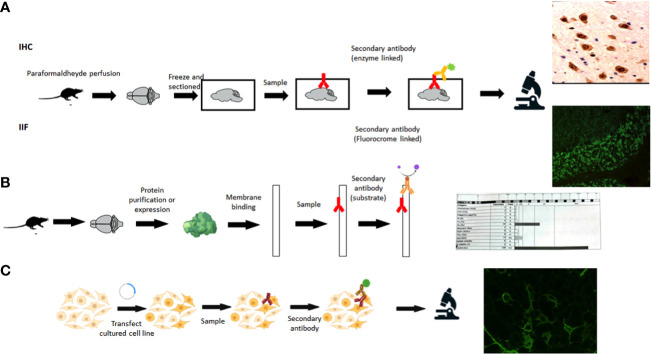
Techniques for autoantibody detection. **(A)** Tissue-based assays, IHC, immunohistochemistry; IIF, indirect immunofluorescence. **(B)** Immunoblot. **(C)** Cell-based assay. (Created in BioRender.com).

Recommended laboratory diagnostics follow a two steps strategy. First, a screening technique (IIF or IHC) should be performed on animal neural tissue (tissue-based assays, TBA) to detect possible reactivity and identify a characteristic pattern, if present (**Figure 1A**).

Screening for intracellular antibodies is usually performed on fixed, permeabilized, cerebellum sections, as the intracellular antigen has to be accessible to autoantibodies (19). Surface antibodies are usually studied on rodent brain sections, lightly fixed and not necessarily permeabilized, allowing protein conformation maintenance.

A confirmatory analysis should then be performed in search of specific autoantibodies (7) with different methodologies accordingly to the type of antibody investigated (antibodies against intracellular vs. cell surface antigens) (20, 21) (**Figure 1B, C**). The type of antibody investigated will depend on the clinical syndrome and on the pattern observed on the tissue.

Specific confirmatory assays for intracellular antibodies usually rely on commercial blots, with purified or recombinant antigens bound to a nitrocellulose membrane (**Figure 1C**). Western Blot (WB) provides an easy and rapid detection of intracellular antibodies in routine laboratories (22). This technique has the advantage that they can be automated and several antigens can be analyzed at the same time. Of note, commercial WB uses recombinant proteins that may not have the conformational structure of the natural protein *in vivo*, epitopes can be recognized by nonspecific antibodies that may be present in patients with other autoimmune or inflammatory diseases (23). Thus, there is a risk of false positive results, which are inversely associated with band intensity (24). A clinical correlation is crucial to avoid false diagnosis (25). In one study carried out on intracellular antibodies, just 30.1% and 36.7% of antibodies detected in commercial inmunoblot of EUROLINE or PNS+2 were confirmed by in-house IIF (24).

Several studies have pointed that when a paraneoplastic syndrome (PNS) is suspected (frequently associated with intracellular antigens), both TBA and immunoblot should be performed (26). If antibody testing relies only on immunoblot, at least positive results must be confirmed by another technique. In these studies, patients with concordant results between immunoblot and TBA (IIF or IHC), were more likely to have PNS and cancer than patients with discordant results. On the other hand, most of the non-confirmed results with immunoblot have an incompatible clinical presentation regarding the antibody identified or an alternative final diagnostic. When analysing bands intensity, samples with strong reacting bands are more likely to be from patients with PNS and also associate with positive TBA, compared with samples with low intensity bands, irrespective of the identified antibody (24, 26). Thus, although immunoblots are more sensitive in detecting low levels of antibodies, they are also less specific than IHC for PNS. Indeed cancer-associated neural antibodies can be found in patients with cancer and no PNS and in healthy individuals at low levels, thus they are no longer referred to as onconeural antibodies but classified into “high risk” and “intermediate risk” for cancer antibodies (9).

Surface antibodies are studied using cell-based assays (CBAs), in which cells lines are transfected with the target protein providing the conformational antigen (**Figure 1C**), this being the gold standard for neuronal surface antibodies (27). Most patients with surface antibodies have antibodies present in the cerebrospinal fluid (CSF), thus the gold standard is to test paired serum and CSF samples to achieve the highest sensitivity and specificity and avoid false-positive and false negative test results (8, 28, 29).

An exception to this rule would be the determination of antibodies against glycine receptor (GlyR) and anti-MOG (from the group of antibodies against glial cells). These antibodies are not detected by immunohistochemical techniques on rat or mouse tissue and must be analyzed directly by immunofluorescence on HEK293 or similar cells transfected with the antigen. On the other hand, anti-AQP4 antibodies (also from the group of antibodies against glial cells) are also detected on transfected cells but are also visualized on rodent tissue. Of note, in some cases, CBAs can improve the detection of some intracellular antibodies, such as CV2 or SOX1 (30) and may be used as confirmatory test instead of WB (24).

**Table 1:** Detection of antineuronal antibodies (very rare antibodies not included) and other antineural antibodies.

Neural intracellular antigens					
*Antibody*	Preferred sample*	*Rodent brain IHC or IIF for intracellular proteins*	*Immunoblot or WB*	*Cell-based assay*	*Other*
Hu	S	**√**	**√**	**(√**)	
Yo	S	**√**	**√**	**(√)**	
Ri	S	**√**	**√**	**(√)**	
CV2/CRMP5	S	**√**	**√**	√**	
Ma2	S	**√**	**√**	**(√)**	
Tr/DNER^&^	S	**√**	**√**	**√**	
Amphiphysin	S	**√**	**√**	N/A	
SOX1	S	(**√)**	**√**	√**	
KLHL11	S	(**√)**	**(√)**	**√**	
GAD¶	CSF>S	**√**	**√**	**√**	ELISA; RIA
AK5	S	**√**	**(√)**	**√**	
GFAP	CSF	**√**	**(√)**	**√**	
**Neural cell surface antigens**					
*Antibody*	Preferred sample*	*Rodent brain IHC or IIF for surface proteins*	*Hippocampal live neurons*	*Cell-based assay*	*Other*
NMDAR	CSF>S	**√**	**√**	**√**	
AMPAR	CSF>S	**√**	**√**	**√**	
GluK2	CSF>S	**√**	**√**	**√**	
GABAaR	CSF>S	**√**	**√**	**√**	
GABAbR	CSF>S	**√**	**√**	**√**	
mGluR1	CSF>S	**√**	**√**	**√**	
mGluR5	CSF>S	**√**	**√**	**√**	
GlyR	CSF > S	X	?	**√**	
LGI1	CSF + S	**√**	**√**	**√**	
Caspr2	CSF + S	**√**	**√**	**√**	
DPPX	CSF>S	**√**	**√**	**√**	
IgLON5	CSF+S	**√**	**√**	**√**	
VGCC	S (CSF)?	X	√***	N/A	RIA
Aquaporin4	S	**√**	N/A	**√**	
MOG	S	X	N/A	**√**	

**√,** Detected; (**√),** May be detected; X, Negative; ?, Unknown; RIA, radioimmunoassay; ELISA, Enzyme-linked immunosorbent assay; N/A, not applicable; WB, Western Blot; CBA, Cell based assay; IHC, immunohistochemistry; IIF, indirect immunofluorescence.

*In most of the antibodies CSF and serum are positive unless only one of them is indicated. CSF>S = indicates that CSF is more disease-specific than serum, or that serum may offer false results. CSF + S= indicates that both are equally recommended.

** CBA to be done when one of the standard tests (IHC or immunoblot) are negative.

*** mild.

¶ Low titers not detected by immunohistochemistry are of no clinical value.

&: included among intracellular antigens because detection is by antibody detection techniques against intracellular antigens.

Regarding antibodies against nodal and paranodal antigens, such as neurofascin-155 and contactin-1, these are IgG4-type and can be identified in a small subgroup of patients with polyradiculoneuropathy, with specific clinical characteristics. Detection techniques for these antibodies include transfected cells, which are usually used for screening and ELISA, WB or IHC on nerve fibers for confirmation (14, 31).

In summary, routine diagnostic laboratories follow established Diagnostic Criteria by the European Network for PNS (Euronetwork) (22) where the objective is a detection with sensitivity (generally IHC or IIF on rodent tissue) and a confirmation with specificity (by immunoblot, WB or CBA). Routine diagnostic laboratories rely only on commercial assays, which are limited to IIF on tissue, and to the available antigens in commercial CBAs and blots (**Table 1**). Commercial kits testing multiple antibodies are helpful, but a number of false positive and negative results exists, particularly for some autoantibodies assessed by blot (26).

Although commercial CBAs are specific, sometimes they are not sensitive enough for some antibodies, such as LGI-1 and GABABR in CSF, and are prone to indeterminate results due to high background reactivity especially of serum samples. Combining TBAs of brain sections with CBAs in case of positive tissue reactivity improves the diagnostic accuracy of testing for autoimmune encephalitis, as shown in several studies, in which the cases with positive brain staining but negative results using commercial kits consisted on cases with antibodies that were missed using the kits but yielded positive results with in-house assays, as well as patients with antibodies to rare antigens not included in the routine panel or patients with antibodies to unknown antigens (30, 32). In addition to patients with antibodies to antigens not included in the commercial CBAs, in house TBAs permit uncharacterized autoantibody detection in some patients fulfilling diagnostic criteria for autoimmune encephalitis (33, 34).

There are some inherent limitations associated with the different assays and commercial kits used, but there is also a lack of standards regarding methodologies and results presentation that should be addressed (9). An unexpected antibody result in the context of the clinical symptoms, type of tumor or patient demographics should raise the possibility of a false positive and be reassessed with different assays and samples or in specialized research laboratories (8, 9).

### Patient samples

Sensitivity and specificity for serum or CSF analysis vary among different antibodies; guidelines therefore recommend to perform antibody testing in both samples (9). For patients with suspicion of disease mediated by antibodies against surface antigens, both serum and CSF must be studied in parallel whenever possible (9, 35) given the different sensitivity and specificity between sample types and across laboratories. Traditionally testing for antibodies against surface antigens has been reported to have a better sensitivity in CSF, with antibodies against NMDA receptor showing better sensitivity when tested in CSF. However, LGI1 and CASPR2 have been reported to show a greater sensitivity with serum testing by some reference laboratories. Furthermore, MOG antibodies, are usually only detected in serum (**Table 1**).

Antibodies may be detected only in the CSF at certain stages of disease or depending on treatments. CSF analysis is particularly important if the results of serum tests do not correspond with the clinical syndrome. In these cases, the study of antibodies in both serum and CSF, can lead to the correct diagnosis, ruling out false positive or false negative results (28).

Furthermore, the analysis of CSF may prove useful to assess for oligoclonal bands or CSF/serum IgG index demonstrating intrathecal synthesis of antibodies, as it can help in determined scenarios, (such as in GAD associated syndromes or seronegative encephalitis) indicating an immune-mediated origin (8).

Taking this into account, and the fact that a lumbar puncture (LP) is an invasive test, provided clinical indications are met (see “when to study neural antibodies” above) CSF should be extracted to allow for analysis of neural antibodies. A tube with at least 12 drops of CSF must be extracted and sent to the Immunology laboratory, always coupled with a sample of the patient’s serum. The samples should preferably be sent before starting immunosuppressive or immunomodulatory treatment, including corticosteroids or immunoglobulins, to prevent this from influencing the results.

All requests for neural antibodies should be accompanied by a brief clinical report stating the clinical presentation including symptoms, onset and timeline. For this purpose, a specific mandatory space should be provided on the request forms for these tests. Given the constant development of this field, we suggest that both serum and CSF from all patients be routinely saved for later analysis. For this, laboratories that study neural antibodies will need freezers at -80 °C for correct conservation of the samples, which should follow an official regularization, in order to have samples that allow for future studies of patients that have not been diagnosed with characterized antibodies, allowing for new determinations in the future as diagnostic techniques advance.

### Referral to reference laboratories

In the case of patients who meet clinical criteria for probable autoimmune encephalitis, or patients with syndromes classically associated with neural antibodies, we suggest that serum and CSF samples be studied in specialized laboratories with experience with these techniques that can guarantee a correct study and result interpretation. Currently, recommendation is to disregard neuronal IgM and IgA antibodies as diagnostic biomarkers; as only IgG antibodies have diagnostic significance (36). Also, unexpected antibody results based on the type of neurologic phenotype, associated tumor, or patient’s age and sex should raise concern for false-positive results and be reassessed with additional studies (9).

When a diagnosis is not achieved, either because no antibody is identified albeit a medium to high suspicion, or because laboratory results are not in line with the patient’s symptoms or clinical characteristics, it would be helpful that laboratories be supported by national reference laboratories, with expertise in clinical diagnosis and investigation into new antibodies.

## Final considerations

**Figure 2:**
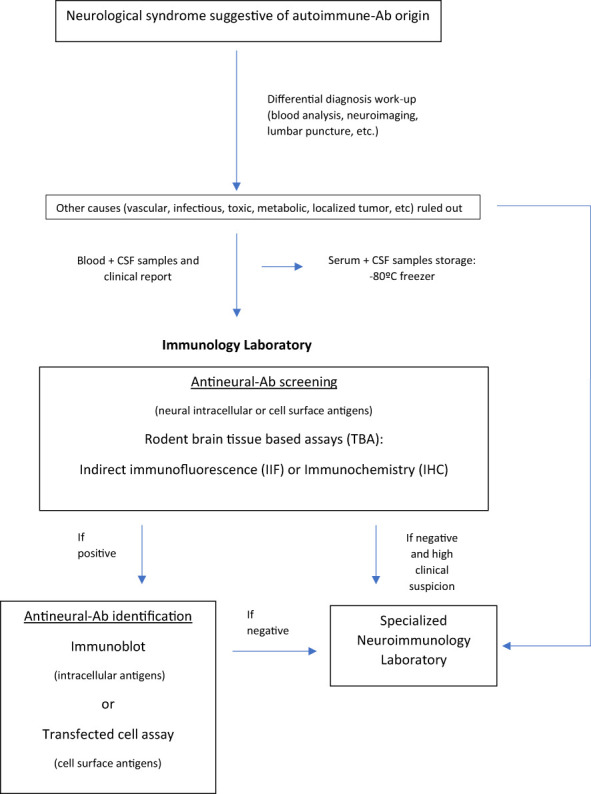
Study of antineural antibodies: work-flow diagram. Ab, antibody; CSF, Cerebrospinal fluid.

In this article we summarize and explain current diagnostic tests of use for the study of neural antibodies, including indications, limitations and pitfalls. We also propose a work flow at a local level, with the possibility of referral to specialised centres, summarised in (**Figure 2**), to facilitate the task of the laboratories involved and to improve the diagnostic process of these patients.

## Author contributions

MF-F: Manuscript conceptualization, Investigation, Writing - Original Draft. LL: Investigation, Writing - Original Draft. NP: Investigation, Writing - Original Draft. CJL: Writing - Review & Editing TBA: Writing - Review & Editing. O-OL: Writing - Review & Editing. IC: Writing - Review & Editing. AC: Manuscript conceptualization, Investigation, Writing - Review & Editing. All authors contributed to the article and approved the submitted version.
